# Immune-Related Gene Expression Profile in Laboratory Common Marmosets Assessed by an Accurate Quantitative Real-Time PCR Using Selected Reference Genes

**DOI:** 10.1371/journal.pone.0056296

**Published:** 2013-02-25

**Authors:** Yoshiki Fujii, Kazutaka Kitaura, Takaji Matsutani, Kenji Shirai, Satsuki Suzuki, Tomohiko Takasaki, Kenichi Kumagai, Yoshie Kametani, Takashi Shiina, Shuji Takabayashi, Hideki Katoh, Yoshiki Hamada, Ichiro Kurane, Ryuji Suzuki

**Affiliations:** 1 Department of Rheumatology and Clinical Immunology, Clinical Research Center for Allergy and Rheumatology, Sagamihara National Hospital, National Hospital Organization, Kanagawa, Japan; 2 Department of Virology 1, National Institute of Infectious Diseases, Tokyo, Japan; 3 Laboratory of Immune Regulation, Wakayama Medical University, Osaka, Japan; 4 Section of Biological Science, Research Center for Odontology, Nippon Dental University, School of Life Dentistry, Tokyo, Japan; 5 Department of Oral and Maxillofacial Surgery, School of Dental Medicine, Tsurumi University, Kanagawa, Japan; 6 Department of Immunology, Division of Basic Medical Science and Molecular Medicine, Tokai University School of Medicine, Kanagawa, Japan; 7 Department of Molecular Life Science, Division of Basic Medical Science and Molecular Medicine, Tokai University School of Medicine, Kanagawa, Japan; 8 Experimental Animals Institute, Hamamatsu University School of Medicine, Shizuoka, Japan; 9 Laboratory of Animal Breeding and Genetics, Central Institute for Experimental Animals, Kawasaki, Japan; Universidad Pablo de Olavide, Centro Andaluz de Biología del Desarrollo-CSIC, Spain

## Abstract

The common marmoset (*Callithrix jacchus*) is considered a novel experimental animal model of non-human primates. However, due to antibody unavailability, immunological and pathological studies have not been adequately conducted in various disease models of common marmoset. Quantitative real-time PCR (qPCR) is a powerful tool to examine gene expression levels. Recent reports have shown that selection of internal reference housekeeping genes are required for accurate normalization of gene expression. To develop a reliable qPCR method in common marmoset, we used *geNorm* applets to evaluate the expression stability of eight candidate reference genes (*GAPDH*, *ACTB*, *rRNA*, *B2M*, *UBC*, *HPRT*, *SDHA* and *TBP*) in various tissues from laboratory common marmosets. *geNorm* analysis showed that *GAPDH*, *ACTB*, *SDHA* and *TBP* were generally ranked high in stability followed by *UBC*. In contrast, *HPRT*, *rRNA* and *B2M* exhibited lower expression stability than other genes in most tissues analyzed. Furthermore, by using the improved qPCR with selected reference genes, we analyzed the expression levels of CD antigens (CD3ε, CD4, CD8α and CD20) and cytokines (IL-1β, IL-2, IL-4, IL-5, IL-6, IL-10, IL-12β, IL-13, IFN-γ and TNF-α) in peripheral blood leukocytes and compared them between common marmosets and humans. The expression levels of CD4 and IL-4 were lower in common marmosets than in humans whereas those of IL-10, IL-12β and IFN-γ were higher in the common marmoset. The ratio of Th1-related gene expression level to that of Th2-related genes was inverted in common marmosets. We confirmed the inverted ratio of CD4 to CD8 in common marmosets by flow cytometric analysis. Therefore, the difference in Th1/Th2 balance between common marmosets and humans may affect host defense and/or disease susceptibility, which should be carefully considered when using common marmoset as an experimental model for biomedical research.

## Introduction

The common marmoset (*Callithrix jacchus*) is a New World monkey and is considered potentially useful as an experimental animal model in research fields such as drug toxicology [1], [2], neuroscience [3], [4], autoimmune diseases [5], [6] and infectious diseases [7], [8], because of its size, availability and high genetic similarity with humans [9], [10]. Compared with mice, common marmosets are more useful as an *in vivo* model to study immune function [11]. However, essential tools and gene information for conducting studies using common marmosets are in short supply or unavailable. For example, monoclonal antibodies specific for common marmosets have been only partially established. Although DNA microarray research for common marmoset brain has been reported [12], sufficient studies have not been performed in other research fields.

Quantitative real-time polymerase chain reaction (qPCR) is the dominant quantitative technique for gene expression analysis due to its broad dynamic range, accuracy, sensitivity, specificity and speed [13]. Thus, qPCR is very useful for investigating physiological and pathological status from a small amount of sample. Normalization to reference genes such as housekeeping genes is usually required for qPCR analysis. However, expression levels of reference genes may vary between tissues, cell types and experimental conditions. Therefore, the validation of suitable reference genes in each experiment is critical for the accurate evaluation of qPCR data. Recently, a set of guidelines for evaluating qPCR experiments was developed [14] and a strict method for the selection of reference genes suitable for normalization was proposed [15]. A freely available program, *geNorm* applet (http://medgen.ugent.be/~jvdesomp/genorm/), can determine gene stability ranking and the number of reference genes required for normalization in a given panel of samples [15].

To develop an accurate and reliable qPCR method for common marmosets, we examined the expression stabilities of candidate reference genes in various tissues of laboratory common marmosets using *geNorm* applet. Then, we compared expression levels of immune-related genes in peripheral blood leukocytes between common marmosets and humans. To the best of our knowledge, this is the first such study for the selection of reference genes in common marmosets. The present data will contribute to future studies of gene expression analysis by qPCR for common marmosets.

## Materials and Methods

### Ethics statement

The study was conducted in accordance with the Act on Welfare and Management of Animals of Japanese government. All animals were housed, cared for, and used according to the principles set forth in the Guide for the Care and Use of Laboratory Animals: Eighth Edition (National Research Council, 2011). All experiments using common marmosets were approved by the committee for animal experiments at the National Institute of Infectious Diseases (Approval Number: 610,007). For humans, whole blood was obtained from eight healthy volunteers (mean age ± sd: 35.7±13.0 years old) after obtaining written informed consent. This study and the consent procedure were approved by the ethics committee of Tokai University School of Medicine (Approval Number: 10I-22).

### Animals

Eight common marmosets (1.58±0.29 years old) were obtained from CLEA Japan, Inc. (Tokyo, Japan) and maintained in specific pathogen-free conditions at the National Institute of Infectious Diseases (Tokyo, Japan). Common marmosets were housed solely or in pairs in a single cages 39 cm (W)×55 (D)×70 (H) in size on 12∶12 h light/dark cycles. Room temperature and humidity were maintained at 26–27°C and 40–50%, respectively. Filtered drinking water was delivered by an automatic watering system and total 40–50 g/individual of commercial marmoset chow (CMS-1M, CLEA Japan) were given in a couple of times per day. Dietary supplements (sponge cakes, eggs, banana pudding, honeys, vitamin C and D3) were also given to improve their health status. Machinery noise and dogs' barks were avoided to reduce stress. The cages were equipped with resting perches and a nest box as environmental enrichment. The marmosets were routinely tested to assure the absence of pathogenic bacteria, viruses, and parasite eggs in the animal facilities and did not exhibited abnormal external appearances. Four common marmosets were euthanized by cardiac exsanguinations under anesthesia with Ketamine hydrochroride (50 mg/kg, IM) and Xylazine (3.0 mg/kg, IM). After sacrifice, various tissues removed, and whole blood was obtained from all eight common marmosets.

### RNA isolation

Heparinized venous blood samples from common marmosets were obtained before sacrifice and incubated in erythrocyte lysis buffer (155 mM NH_4_Cl, 10 mM KHCO_3_, and 0.1 mM EDTA). Following incubation on ice for 5 min, cells were centrifuged at 300×*g* for 10 min at 4°C and washed with lysis buffer and then PBS. Leukocytes were lysed with QIAzol® Lysis Reagent (Qiagen, Hilden, Germany) and total RNA was extracted using an RNeasy® Plus Universal Mini Kit (Qiagen) according to the manufacturer's instructions. Tissue samples (spleen, mesenteric lymph node, jejunum, ileum, descending colon, cerebrum, cerebellum, brainstem, heart, lung, liver and kidney) were excised from each animal and immediately submerged in RNAlater® RNA Stabilization Reagent (Qiagen). Then total RNA was extracted using RNeasy® Plus Universal Mini Kit (Qiagen). RNA concentration and integrity were assessed using the Agilent RNA 6,000 Nano Kit (Agilent Technologies, Inc., CA, USA) in an Agilent 2100 Bioanalyzer. All RNA samples were confirmed to have no degradation and were of optimal quality for downstream qPCR applications.

### Candidate reference genes

Based on a literature search, eight commonly used candidate internal control genes were selected for analysis: *GAPDH* (glyceraldehyde-3-phosphate dehydrogenase), *ACTB* (actin, beta), *rRNA* (18S ribosomal RNA), *B2M* (beta-2-microglobulin), *UBC* (ubiquitin C), *HPRT* (hypoxanthine phosphoribosyltransferase 1), *SDHA* (succinate dehydrogenase complex, subunit A) and *TBP* (TATA-box binding protein). All genes chosen have independent cellular functions and are not thought to be co-regulated. The sequences of primers specific for each reference gene are shown in Table 1.

**Table 1:** Sequences of qPCR primers for housekeeping genes.

Target gene	Species	5′-primer sequence -3′ a) ^,^ b)		Product size (bp)	PCR efficiency	Reference
		Forward	Reverse			
GAPDH	Cj	TCGGAGTCAACGGATTTGGTC	TTCCCGTTCTCAGCCTTGAC	181	0.920	DD279474
	Hs	---------------------	--------------------	181	0.921	AF261085
ACTB	Cj	GATGGTGGGCATGGGTCAGAA	AGCCACACGCAGCTCGTTGT	163	0.901	DD279463
	Hs	---------------------	---------------A----	163	0.883	NM_001101
HPRT	Cj	ATCCAAAGATGGTCAAGGTCG	GTATTCATTATAGTCAAGGGCATA	134	0.842	DD289567
	Hs	---------------------	------------------------	134	0.880	M31642
B2M	Cj	CTATTCAGCATGCTCCAAAGA	AAGACAAGTCTGAATGCTCCAC	168	0.928	AF084623
	Hs	----C----G-A---------	----------------------	168	0.950	AB021288
UBC	Cj	TCCCTTCTCGGCGGTTCTG	. TGCATTGTCAAGCGGCGAT	158	0.922	AB571242
	Hs	-------------A-----	TC----------T-A----	160	0.936	NM_021009
rRNA	Cj	CGACCATAAACGATGCCGAC	GGTGGTGCCCTTCCGTCAAT	145	0.918	AB571241
	Hs	--------------------	--------------------	145	0.940	M10098
SDHA	Cj	TGGGAACAAGAGGGCATCTG	CCACCACGGCATCAAATTCATG	86	0.934	XM_002745154
	Hs	--------------------	-------T--------------	86	0.948	BC001380
TBP	Cj	CCATGACTCCCGGAATCCCTAT	ATAGGCTGTGGGGTCAGTCCA	70	0.920	EU796973
	Hs	----------------------	---------------------	70	0.954	M55654

a) Hyphen indicates a nucleotide identical to human sequences.

b) Dot indicates a shift nucleotide to marmoset sequences.

### Quantitative real-time PCR

First-strand cDNA was synthesized using PrimeScript® RT reagent Kit (Takara Bio, Otsu, Japan) with attached random hexamers and oligo(dT) primers. Reactions were incubated at 37°C for 15 min followed by 85°C for 5 sec according to the manufacturer's instructions. Then each cDNA sample was diluted with RNase/DNase-free water to 25 ng/µL. The expression level of each gene was analyzed by qPCR using the Bio-Rad CFX96 system (Bio-Rad Laboratories, Inc., Hercules, CA, USA). PCR reactions consisted of 5 µL of SsoFast™ EvaGreen® Supermix (Bio-Rad), 3.5 µL of RNase/DNase-free water, 0.5 µL of 5 µM primer mix, 1 µL of cDNA in a total volume of 10 µL. The primer sequences are shown in Tables 1 and 2. Cycling conditions were as follows: 30 sec at 95°C followed by 45 rounds of 95°C for 1 sec and 60°C for 5 sec. Melting curve analysis to determine the dissociation of PCR products was performed between 65°C and 95°C. Data were expressed as mean values of experiments performed in triplicate. Seven points of a 10-fold serial dilution of standard DNA was used for absolute quantification. Standard DNA was generated by cloning PCR products into pGEM-T Easy Vector (Promega, WI, USA). Sequences of the cloned plasmid were confirmed by DNA sequencing using the CEQ8000 Genetic Analysis System (Beckman Coulter). Quality and concentration of the plasmid DNA were validated using Agilent DNA 7,500 Kit in an Agilent 2100 Bioanalyzer.

**Table 2:** Sequences of qPCR primers for CD markers and cytokines.

Target gene	Species	5′-primer sequence -3′ a) , b)		Product size (bp)	PCR efficiency	Reference
		Forward	Reverse			
CD3ε	Cj	GGCTTGCTGCTGCTGGTTTAC	CCGGATGGGCTCATAGTCTG	150	0.865	DQ189218
	Hs	---------------------	--------------------	150	0.848	NM_000733
CD4	Cj	GGAAAACGGGAAAGTTGCATCA	GCCTTCTCCCGCTTAGAGAC	163	0.926	AF452616
	Hs	C------A--------------	--------------C-----	162	0.907	M35160
CD8α	Cj	TCTCCCAAACCAAGTCCAAGG	AGTTTCTCAGGGCCGAGCAG	144	0.940	DQ189217
	Hs	---------A----C------	. ---G---------------	143	0.912	NM_001768
CD20	Cj	GGGCTGTCCAGATTATGAATG	GAGTTTTTCTCCGTTGCTGC	166	0.942	DQ189220
	Hs	---------------------	--------------------	166	1.002	X07203
IL-1β	Cj	TGCACCTGTACGATCCCTGAAC	TTGCACAAAGGACATGGAGAACAC	145	0.806	AB539804
	Hs	---------------A------	---T--------------------	145	0.780	NM_000576
IL-2	Cj	CCCAAGAAGGCCAAAGAATTG	CTTAAGTGAAAGTTTTTGCTTTGAG	104	0.773	DQ826674
	Hs	-------------C----C--	-------------------------	103	0.797	BC070338
IL-4	Cj	CATTGTCACAGAGCAAAAGACTC	CTCAGTTGTGTTCTTGGAGGCA	79	0.910	XM_002744606
	Hs	. GCC----------G-------	----------------------	77	0.878	NM_000589
IL-5	Cj	AATCACCAACTGTGCACTGAAGAA	. TTTGGCGGTCAATGTGTTCCTT	130	0.871	DQ658152
	Hs	------------------------	TT------C--------A--T---	132	0.860	NM_000879
IL-6	Cj	GATTCAATGAGGAGACTTGCC	TGTTCTGGAGGTACTCTAGGTA	81	0.920	DQ658153
	Hs	---------------------	----------------------	81	0.990	NM_00600
IL-10	Cj	CTGCCTCACATGCTTCGAGA	TGGCAACCCAGGTAACCCTTA	134	0.970	DQ658154
	Hs	------A-------------	---------------------	134	0.920	M57627
IL-12β	Cj	. GGACGGCAAGGAGTATGAGTA	TTGAGCTTGTGAACGGCATC	111	0.935	AB539805
	Hs	G----AA---------------	--------------------	112	0.900	M65272
IL-13	Cj	TCCAGCTTGCTTGTCCGAG	CTGCAAATAATGATGCGTT-GATGT	127	0.916	AB571243
	Hs	----------A--------	. ---------------T--C--A--	127	0.964	NM_002188
IFN-γ	Cj	GGGTTCTCTTGGCTGTTACTG	TGTCTAAGAAAAGAGTTCCATTATC	116	0.838	FJ598593
	Hs	---------------------	. -C----------------------	115	0.856	NM_000619
TNF-α	Cj	AGCCTGTAGCCCATGTTGTAG	CTCTCAGCTCCACGCCATTG	102	0.887	DQ520835
	Hs	---------------------	--------------------	102	0.817	NM_000594

a) Hyphen indicates a nucleotide identical to human sequences.

b) Dot indicates a shift nucleotide to marmoset sequences.

### Analysis of gene expression stability

The expression stability of selected reference genes was evaluated using a publicly available program, *geNorm* applet [15]. *geNorm* calculates the stability of tested reference genes according to the similarity of their expression profiles by pairwise comparison and M value, where the gene with the highest value is the least stable one. It is possible to perform sequential elimination of the least stable gene in any given experimental group, thus resulting in the exclusion of all but the two most stable genes in each case.

### Flow cytometry

Heparinized peripheral blood was collected from common marmosets and centrifuged in Lymphocepal (IBL Co. Takasaki, Japan) at 2,000 rpm for 30 min. Mononuclear cells were collected and re-suspended in RPMI1640 medium containing 10% fetal calf serum. Cells were stained with anti-common marmoset CD8 antibody (Mar8–10) [16] for 15 min at 4°C and washed with 1% (w/v) bovine serum albumin-containing PBS. Subsequently, cells were stained with phycoerythrin-labeled secondary antibody, peridinin chlorophyll protein cyanin5.5 (PerCPCy5.5)-conjugated anti-human CD3 (SP34-2) and Alexa488-conjugated anti-common marmoset CD4 (Mar4-33) antibodies [16]. Peripheral blood from healthy human volunteers was collected and mononuclear cells isolated by Ficoll-Paque (GE Healthcare Biosciences, Uppsala, Sweden) gradient centrifugation. The monoclonal antibodies used for cell staining were as follows: PerCPCy5.5-conjugated anti-human CD3 (SP34-2), allophycocyanin-conjugated anti-human CD4 (SK3), fluorescein isothiocyanate-conjugated anti-human CD8 (HIT8a) (BD PharMingen). Cells were analyzed by FACSCalibur (Becton Dickinson, Franklin Lakes, NJ, USA).

### Statistical analysis

Student's *t*-test was used for statistical analysis to assess significant differences in qPCR assays. A *P* value<0.05 was considered to be statistically significant.

## Results

### The expression levels of candidate reference genes in tissues

**Figure 1:**
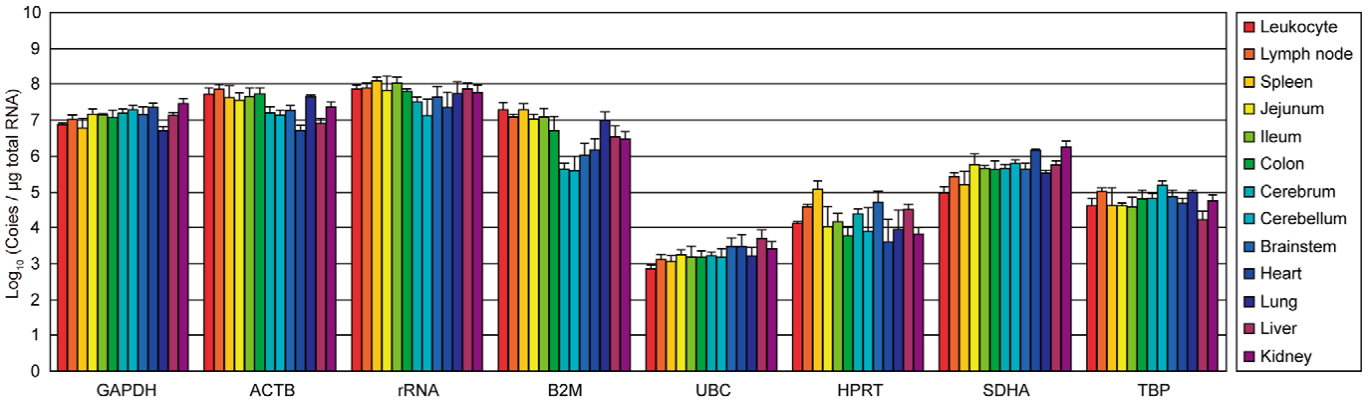
Absolute copy numbers of candidate reference genes. The expression level of each gene in 13 tissues is shown as a logarithmic histogram of absolute copy numbers per µg of total RNA. Means and standard deviations of four individuals are indicated. GAPDH: glyceraldehyde-3-phosphate dehydrogenase; ACTB: actin, beta; rRNA: 18S ribosomal RNA; B2M: beta-2-microglobulin; UBC: ubiquitin C; HPRT: hypoxanthine phosphoribosyltransferase 1; SDHA: succinate dehydrogenase complex, subunit A; TBP: TATA-box binding protein.

Eight housekeeping genes were chosen as reference genes: *GAPDH*, *ACTB*, *rRNA*, *B2M*, *UBC*, *HPRT*, *SDHA* and *TBP*. We determined the transcription levels of these eight genes in 13 tissues (leukocyte, spleen, lymph node, jejunum, ileum, colon, cerebrum, cerebellum, brainstem, heart, lung, liver and kidney) from four individual common marmosets by qPCR. The sequences of primers specific for each reference gene are shown in Table 1. The expression level of each gene in each tissue is shown as the copy number per µg of purified total RNA (Figure 1). The most abundant gene was *rRNA* while the rarest gene was *UBC* and the difference in expression level between the two genes was more than 100,000-fold. For several genes, the expression levels were highly different among tissues. For example, *B2M* expression in heart and brain segments (cerebrum, cerebellum and brainstem) was markedly lower than in other tissues. *HPRT* expression also showed a large variability among tissues. In addition, the expression levels of *rRNA*, *B2M* and *HPRT* varied among individuals; the mean values of standard deviation were 0.224, 0.235 and 0.303, respectively, while those of the other genes were below 0.2.

### A variety of gene expression stabilities among tissues

To evaluate the expression stability of selected reference genes, we used a publicly available program, *geNorm* applets. *geNorm* provides a ranking of tested genes based on the reference gene stability measure M, which is defined as the average pairwise variation of a particular gene compared with all other control genes. Thus, genes with higher M values have greater variations of expression. In addition, assessment of the pairwise variations (V_n/n+1_) between each combination of sequential normalization factors allows identification of the optimal number of reference genes. In the original publication describing *geNorm* [15], a threshold of 0.15 for pairwise variation was established, below which the inclusion of additional reference genes was not necessary.

**Figure 2:**
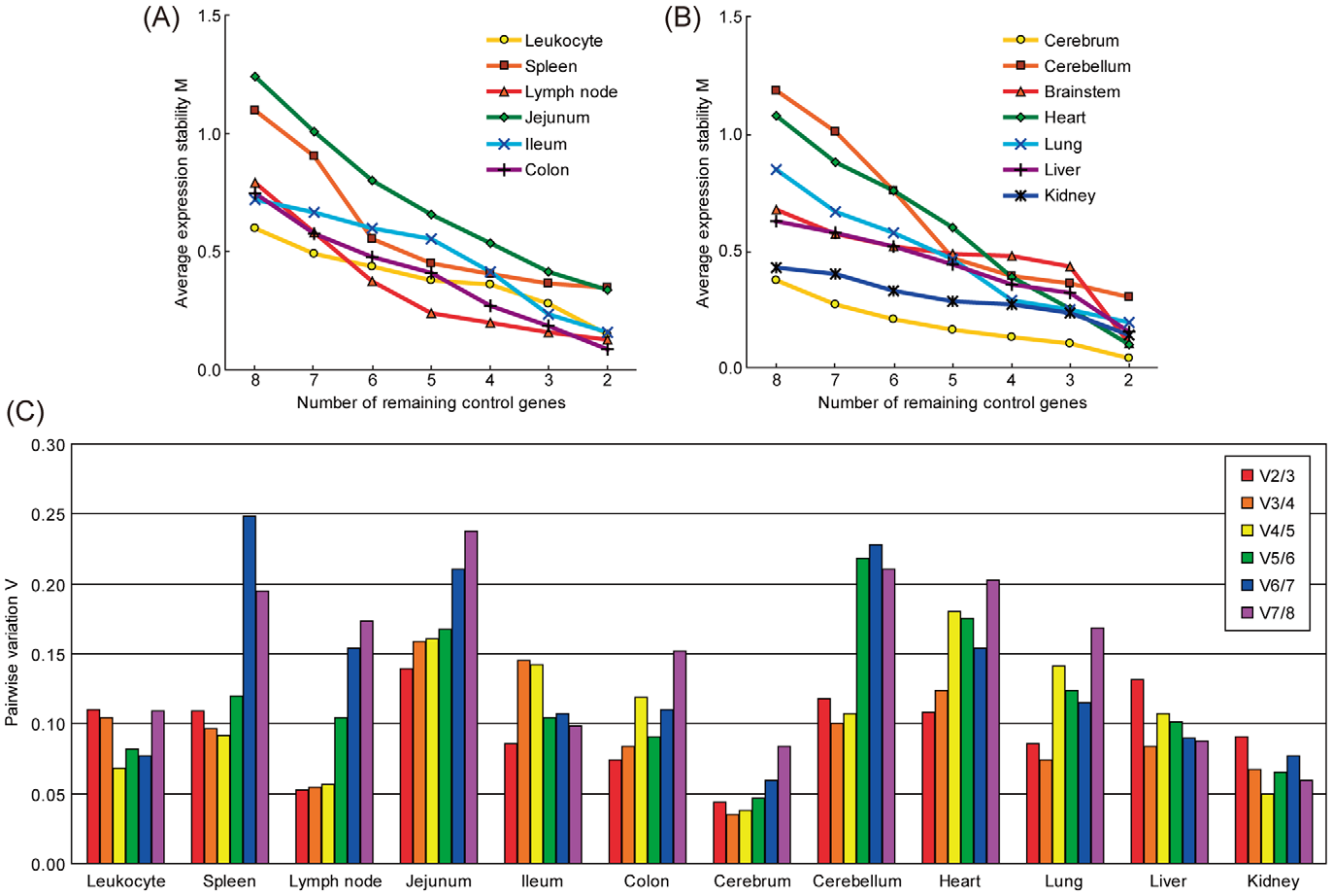
Gene expression stability and pairwise variation of candidate reference genes using *geNorm* analysis. (A) and (B): Average gene expression stability values M of the remaining reference genes during stepwise exclusion of the least stable gene in the different tissue panels are shown. Data are divided into two figures to avoid closely-packed lines. See also figure 3 for the ranking of genes according to their expression stability. (C) Pairwise variation analysis was used to determine the optimal number of reference genes for use in qPCR data normalization. The recommended limit for V value is 0.15, the point at which it is unnecessary to include additional genes in a normalization strategy.

**Figure 3:**
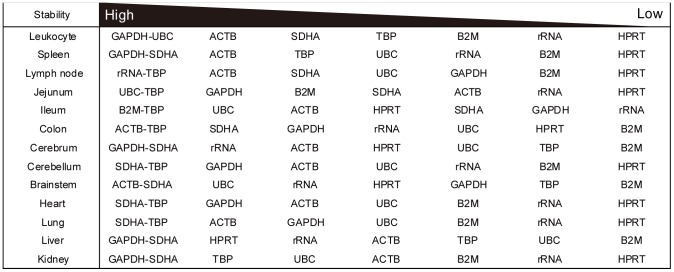
Ranking of gene expression stability of candidate reference genes using *geNorm* analysis. Candidate reference genes are ranked in order of stability for each tissue with the two most stable genes at the left and the least stable at the right.

*geNorm* analysis produced line plots indicating the mean expression stability M of the remaining candidate reference genes in each round of the analysis (Figure 2A and 2B), the pairwise variation V (Figure 2C) and ranking of the candidate reference genes from the least stable to the two most stable genes (Figure 3). The stability score M indicated that gene expression in spleen, jejunum and cerebellum were relatively less stable than other tissues (Figure 2A and B). However, all tissues tested exhibited high stabilities, as M values were less than 1.5, which was the default limit even when all eight genes were analyzed. According to pairwise variation V (Figure 2C), the two most stable genes were sufficient for a stable and valid reference for each tissue analyzed by qPCR because V2/3 values were less than 0.15 in all tissues. Jejunum was the most variable tissue with a V_2/3_ value of 0.139. Figure 3 shows ranking of gene expression stability based on M values. *GAPDH*, *ACTB*, *SDHA* and *TBP* had higher stability, while *HPRT*, *rRNA* and *B2M* were variable in most tissues. *TBP* in intestinal segments (jejunum, ileum and colon) and *SDHA* in brain segments (cerebrum, cerebellum and brain stem) were particularly stable. *HPRT* ranked as the worst of the eight genes in the 13 tissues tested.

### Comparison of gene expression levels between human and common marmoset leukocytes

**Figure 4:**
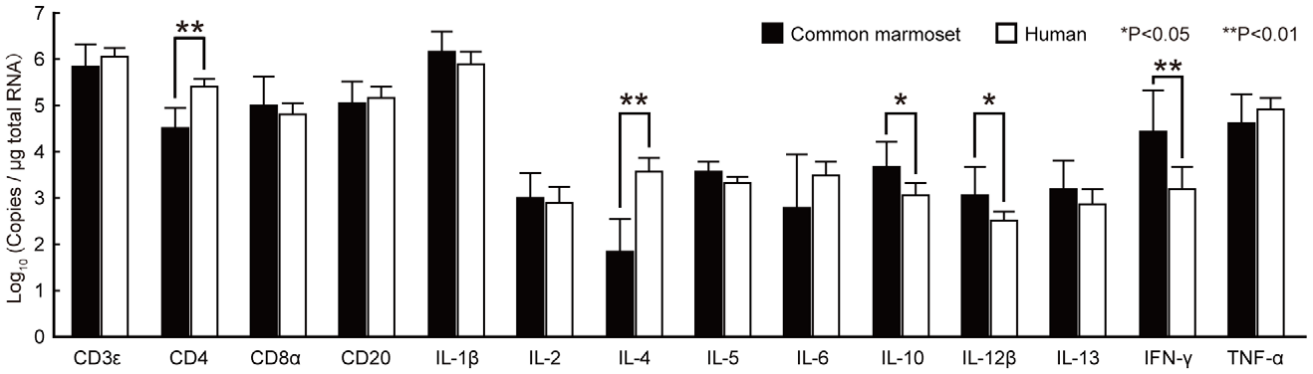
The expression levels of CD antigens and cytokine genes in common marmoset and human leukocytes. The expression level of each gene is shown as a logarithmic histogram of absolute copy numbers per µg of total RNA. Means and standard deviations of eight individuals are indicated. Asterisk indicates statistically significant differences between marmosets and humans by Student's *t*-test (*P value<0.05, **P value<0.01).

Subsequently, we analyzed gene expression levels of four CD antigens (CD3ε, CD4, CD8α, and CD20) and ten cytokines, interleukin (IL)-1β, IL-2, IL-4, IL-5, IL-6, IL-10, IL-12β, IL-13, interferon (IFN)-γ and tumor necrosis factor (TNF)-α, in peripheral blood leukocytes from humans and common marmosets (Figure 4). The sequences of primers specific for these immune-related genes are shown in Table 2. The normalization factor for common marmoset leukocytes was calculated using *GAPDH* and *UBC* based on the *geNorm* analysis as described above. For human leukocytes, we found that the expression of all eight genes were stable (M value = 0.363), of which *ACTB* and *HPRT* had the best score (M value = 0.163, V_2/3_ = 0.062) and were selected for use. The expression levels of CD4 and IL-4 were significantly lower in common marmosets than in humans while those of IL-10, IL-12β and IFN-γ were significantly higher in common marmosets compared with humans. Of interest, the expression level of IL-4 was notably lower in common marmosets than humans, and was close to the detection limit. There was no statistical difference in the expression levels of the other genes tested between common marmosets and humans.

### Difference of CD4/CD8 ratio between humans and common marmosets

**Figure 5:**
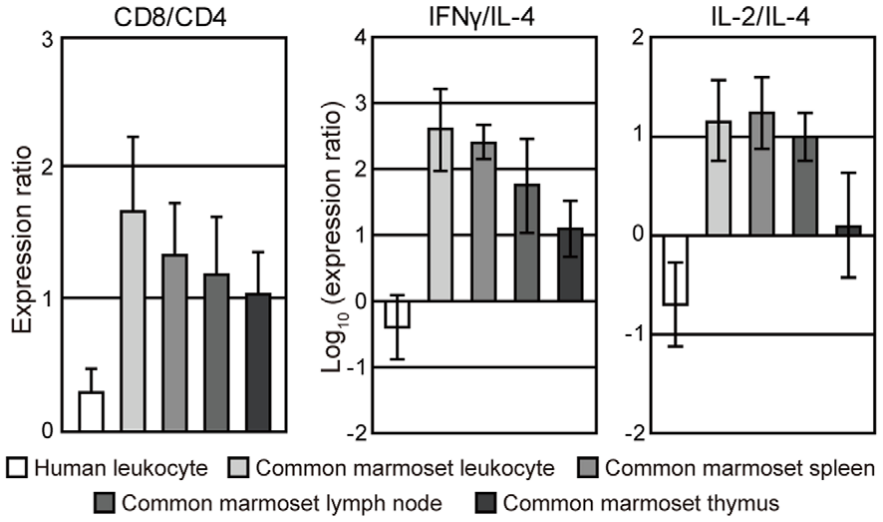
The expression ratios of CD8 to CD4 (CD8∶CD4) and Th1-related genes to Th2-related genes. The ratio of CD8∶CD4 (left panel), IFN-γ∶IL-4 (middle panel) and IL-2∶IL-4 (right panel) in human and common marmoset leukocytes, spleen, lymph node and thymus are shown. Significant differences in the CD8∶CD4, IFN-γ∶IL-4 and IL-2∶IL-4 ratios were found between human leukocytes and common marmoset tissues (*P<0.05).

**Figure 6:**
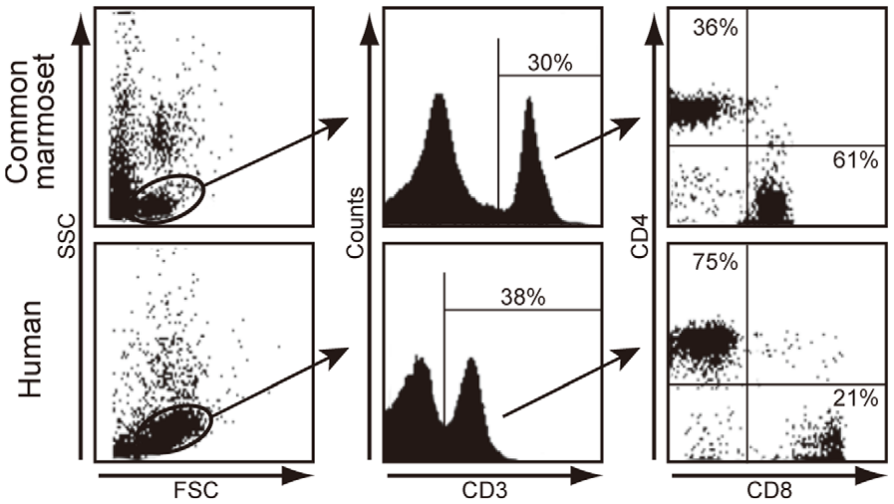
The ratio of CD4^+^ to CD8^+^ cells in common marmoset and human peripheral blood mononuclear cells (PBMCs) by flow cytometry. Representative scattered plots of FSC and SSC are shown in the left panels. Middle panels represent a histogram of CD3 analyzed in the lymphocyte gate. Gated CD3^+^ cells were analyzed for CD4 and CD8 expression (right panels).

We calculated ratios of the expression levels of CD4 to CD8 (CD4/CD8 ratio) in human and common marmoset leukocytes (Figure 5, left panel). CD4/CD8 ratios were significantly higher in human leukocytes compared with common marmoset leukocytes (mean ± sd, 0.59±0.22 vs. −0.49±0.41, *P*<0.01). To confirm the difference in CD4/CD8 ratios, we examined the proportion of CD4^+^ and CD8^+^ in CD3^+^ T cells by flow cytometric analysis. As shown in Figure 6, the rates of CD3^+^ cells in the lymphocyte gate were similar between common marmosets (30%) and humans (38%). However, the rates of CD4^+^/CD3^+^ cells and CD8^+^/CD3^+^ cells was 36% and 61% in common marmosets, respectively, and 75% and 21% in humans, respectively. Similarly, the CD4/CD8 ratio was markedly different between common marmosets and humans (mean ± sd, 0.56±0.08 vs. 3.22±0.35, *P*<0.01) by qPCR. This indicated a good correlation between the results from FACS analysis and that of qPCR analysis. To examine whether the CD4/CD8 ratio is affected by age, we further performed FACS analyses with PBMCs from young and old marmosets (Table 3). The result showed that the inverted CD4/CD8 ratio was fairly constant among individuals and over ages.

**Table 3:** CD8/CD4 ratio in PBMCs from young and old marmosets.

Age	Sex	% positive		CD8/CD4 ratio
		CD8	CD4	
3 month*	male	58.3	38.4	1.52
1.5 year	female	60.7	36.1	1.68
1.5 year*	male	55.1	41.5	1.33
2.0 year	male	52.7	44.6	1.18
10 year*	female	58.6	37.8	1.55
Mean ± sd		57.1±3.2	39.7±3.4	1.45±0.20

* Only FACS analysis, but not qPCR, was done with PBMCs from these three marmosets.

### Difference in T helper 1 (Th1)/T helper 2 (Th2) balance between humans and common marmosets

We compared the ratios of expression levels of Th1-related genes (IFN-γ or IL-2) and Th2-related genes (IL-4) (IFN-γ∶IL-4 or IL-2∶IL-4 ratio) (Figure 5, middle and right panels). Both logarithmic values of the IFN-γ∶IL-4 and IL-2∶IL-4 ratios were negative in human leukocytes whereas those of common marmoset leukocytes, spleen, lymph node and thymus indicated positive values, showing a clear difference in the Th1/Th2 balance between humans and common marmosets.

## Discussion

In the present study, we evaluated the expression stability of common marmoset housekeeping genes in various tissues. To the best of our knowledge, this is the first report of a systematic evaluation of potential reference genes in common marmosets. We chose eight commonly used classical housekeeping genes. Of all genes tested, *rRNA* showed the most abundant expression and *UBC* showed the lowest expression. The *UBC* gene contains multiple directly repeated ubiquitin coding sequences (i.e., polyubiquitin precursor protein) [17]. However, the primer set we used enabled amplification of the unrepeated sequence at the 5′ region of the *UBC* gene only. Thus, low *UBC* expression in our data does not reflect the amount of ubiquitin C protein. *B2M* expression levels were markedly lower in brains and hearts than in other tissues. Resident brain cells normally express few or no MHC class I and B2M molecules [18]–[20]. In addition, *B2M* expression is upregulated by infection or autoimmune disease [21]–[23]. Therefore, in disorders with cellular infiltration such as inflammation (especially encephalitis) or cancer cell invasion, *B2M* expression levels may be significantly varied compared with normal tissue. Thus, we predict that *B2M* may be unsuitable as a reference gene in many cases.

We assessed gene expression stability using the *geNorm* applet. As shown in Figure 2, *geNorm* analysis indicated that all tested genes were stable in each tissue. However, there were some trends in the stability ranking (Figure 3). For example, *TBP* in intestine segments and *SDHA* in brain segments represented prominently high stabilities. *GAPDH*, *ACTB*, *SDHA* and *TBP* were generally ranked high followed by *UBC*. In contrast, the stability of *rRNA* was generally low. This suggests the amount of mRNA is not always proportional to that of total RNA as reported by other studies [24], [25]. In addition, *HPRT*, *rRNA* and *B2M* varied widely among tissues and rarely ranked high.

We analyzed the expression levels of CD antigens and cytokines by qPCR to compare the characteristics of peripheral blood leukocytes between common marmosets and humans (Figure 4). We observed that the expression levels of CD4 and IL-4 were lower in common marmosets than in humans. In contrast, the expression levels of IL-10, IL-12β and IFN-γ were higher in common marmosets. We calculated PCR efficiency of each primer set and found there was no great difference between primers for common marmosets and those for humans (Tables 1 and 2). Thus, the differences in the gene expression levels between common marmosets and humans are not attributable to the differences in PCR efficiency.

We also observed that the CD4∶CD8 ratio and Th1/Th2 balance were inverted in common marmosets by qPCR analysis (Figure 5). In particular, we confirmed the inverted CD4∶CD8 ratio by flow cytometric analysis (Figure 6 and Table 3). The inverted CD4∶CD8 ratio was stable over age. Of interest, we noted that the Th1/Th2 balance is different between common marmosets and humans, although we can only speculate on the cause of the difference. First, intestinal parasite infections may affect the Th1/Th2 balance by regulating expression of genes encoding cytokines [26]–[28]. In particular, protozoan parasites are potent stimulators of IFN-γ expression and Th1 responses [29]. Moreover, humans living in poor hygienic conditions in developing countries had higher Th1 cytokine levels compared with people in developed countries [30]. Although the common marmosets used in this study were maintained in specific pathogen-free conditions, we cannot rule out that such infectious agents may be one of a number of factors responsible for the difference in Th1/Th2 balance.

A second possible reason may be a difference in the number of cells producing the respective cytokines. As shown in Figure 6, the ratio of CD4^+^ to CD8^+^ cells were markedly different in total leukocytes from common marmosets and humans. Since IL-4 is mainly produced by CD4^+^ T cells [31], [32], its expression level may be influenced by the CD4∶CD8 ratio. However, this is not true for all the cytokines tested. For example, the expression levels of IL-2, IL-5 and IL-13, largely produced by T cells, were not significantly different between common marmosets and humans. Therefore, we suggest that the CD4∶CD8 ratio has little effect on Th1/Th2 balance. IL-10 is produced by T cells and monocytes [33] and IL-12β is naturally produced by dendritic cells and macrophages [34], [35]. However, we could not verify these cell numbers in the common marmoset. Further studies are required to determine whether the numbers of cytokine-producing cells influence the expression levels of IL-10 and IL-12β.

Another possibility is genetic variation. Bostik et al., reported distinct sequence differences in the promoter region or the proximal region of cytokine genes including IL-4, IL-10, IL-12β and TNF-γ among humans, macaque and mangabey monkeys, which affected regulation of cytokine synthesis [36]. Jeong et al., reported that the expression level of IL-4 was lower in monkeys (baboon and macaque) than in hominoids (human and chimpanzee) while the expression levels of IL-12β and the IFN-γ were higher in monkeys [37]. It is likely that Th1 dominant expression is common to primates other than hominoids and the difference in Th1/Th2 balance may be caused by genetic differences between common marmosets and humans.

The use of common marmoset is growing in popularity as a non-human primate model in many fields including autoimmune disease and infectious disease. In this study, we presented data regarding gene expression stabilities of common marmoset housekeeping genes and differences in the Th1/Th2 balance between common marmosets and humans. This difference may affect host defense and/or disease susceptibility, which should be carefully considered in biomedical research using common marmoset as an experimental model. We believe our data will contribute to future investigations using common marmoset models of various diseases.
